# Lithium intoxication due to furosemide interaction – a case report

**DOI:** 10.1192/j.eurpsy.2023.1464

**Published:** 2023-07-19

**Authors:** I. Vaz, J. Vitória-Silva

**Affiliations:** 1 Psychiatry, Centro Hospitalar de Trás-os-Montes e Alto Douro, EPE, Vila Real, Portugal

## Abstract

**Introduction:**

Lithium has been used for the management of psychiatric illnesses over the years and it continues to be the first-line mood stabilizer used in treatment and prevention of bipolar disorder. Due to its narrow therapeutic index, other prescribed medications can increase lithium levels and potentiate its toxic effects. Among the most described drugs, non-steroid anti-inflammatory drugs and diuretics (mainly thiazides and loop diuretics) are the most commonly implicated. Risk factors for developing lithium toxicity include old age, polypharmacy, renal impairment, hyponatremic and hypovolemic conditions. Although there is interindividual susceptibility, older patients are at particularly higher risk.

**Objectives:**

To summarize the latest literature about this field and to present a case report as a basis for discussion.

**Methods:**

A brief review of the latest literature was performed, using *PubMed* and the keywords *“lithium”* and *“acute renal injury”.* Also, a case report about a 73 year-old man who suffer from lithium intoxication due to lithium-furosemide interaction is presented.

**Results:**

In the presented case, Mr. F, 73 years old, independent for activities of daily living, was admitted to the Internal Medicine ward due to acute renal injury and lithium intoxication. Initially he was non-collaborative, sleepy, disoriented in all references and speechless. The creatinine was 1.28 mg/dL, urea 63 mg/dL, unspecific leucocitosis 17000/mm^3^, C-Reactive Protein 8 mg/dL, lithium 2.22 mEq/L. The psychiatrist was called to approach the psychiatric status, but as the patient awareness was impaired, the mental state examination was not possible. The patient’s daughter was interviewed. The patient had bipolar disorder (BD) type 1 and was diagnosed with mild cognitive impairment (MCI) a year ago. He had been stable for BD and MCI until the last month. He began to present nocturia, so he went to a urologist who prescribed him furosemide 40 mg daily for benign prostatic hyperplasia (BPH). Since that moment, he started being confused and progressively went to the state that was previously described at the admission of internment. Furosemide, quetiapine and lithium were stopped. He got better, to his previous state, and then started quetiapine 200 mg/day and tansulosine 0.4 mg/day.

**Conclusions:**

Initiating diuretics in patients under lithium should be carefully considered and lithium blood levels must be monitored more regularly when new drugs are prescribed. Other medications must be regarded as alternatives but, if it is not possible, they should be used in the lowest dose and shortest duration as possible. With this case report, we highlight the importance of considering patients as a whole, taking both their physical and mental well-being into account. Healthcare professionals are invited to coordinate their efforts to deliver the best standard of care.

**Disclosure of Interest:**

None Declared

